# Effects of *Saccharomyces cerevisiae* and phytase co-fermentation of wheat bran on growth, antioxidation, immunity and intestinal morphology in broilers

**DOI:** 10.5713/ajas.20.0399

**Published:** 2020-10-13

**Authors:** Wen-Yang Chuang, Li-Jen Lin, Yun-Chen Hsieh, Shen-Chang Chang, Tzu-Tai Lee

**Affiliations:** 1 Department of Animal Science, National Chung Hsing University, Taichung, 402, Taiwan; 2 School of Chinese Medicine, College of Chinese Medicine, China Medical University, Taichung 402, Taiwan; 3 Kaohsiung Animal Propagation Station, Livestock Research Institute, Council of Agriculture, 912, Taiwan; 4 The iEGG and Animal Biotechnology Center, National Chung Hsing University, Taichung, 402, Taiwan

**Keywords:** Wheat Bran, Co-fermented, Phytase, *Saccharomyces cerevisiae*, Postbiotic

## Abstract

**Objective:**

The aim of this study was to investigate the effects of different amounts of wheat bran (WB) inclusion and postbiotics form by *Saccharomyces cerevisiae* and phytase co-fermented wheat bran (FWB) on the growth performance and health status of broilers.

**Methods:**

Study randomly allocated a total of 300 male broilers to a control and 4 treatment groups (5% WB, 5% FWB, 10% WB, and 10% FWB inclusion, respectively) with each pen having 20 broilers and 3 pens per treatment.

**Results:**

The WB does not contain enzymes, but there are 152.8, 549.2, 289.5, and 147.1 U/g dry matter xylanase, protease, cellulase and β-glucanase in FWB, respectively. Furthermore, FWB can decrease nitric oxide release of lipopolysaccharide stimulated chicken peripheral blood mononuclear cells by about two times. Results show that 10% FWB inclusion had significantly the highest weight gain (WG) at 1 to 21 d; 5% FWB had the lowest feed conversion rate at 22 to 35 d; 10% WB and 10% FWB inclusion have the highest villus height and *Lactobacillus* spp. number in caecum; and both 5% and 10% FWB can increase ash content in femurs. Compared to control group, all treatments increase mucin 2, and tight junction (TJ), such as occludin, claudin-1, zonula occludens-1, and mRNA expression in ileum by at least 5 folds. In chicken peripheral blood mononuclear cells, nicotinamide adenine dinucleotide phosphate-oxidase-1 mRNA expression decreases from 2 to 5 times, and glutamate-cysteine ligase catalytic subunit mRNA expression also increases in all treatment groups compared to control group. The mRNA expression of pro-inflammatory cytokines, including interleukin-6 (IL-6), nuclear factor-κB, and IL-1β, decreases in 5% and 10% FWB groups compared to control group.

**Conclusion:**

To summarize, both WB and FWB inclusion in broilers diets increase TJ mRNA expression and anti-oxidation and anti-inflammation, but up to 10% FWB groups have better WG in different stages of broiler development.

## INTRODUCTION

As climate change and global crude prices rise, corn, the main ingredient of polysaccharide source of poultry diet and regarded as one of the materials for biomass energy is becoming more expensive. Furthermore, the outbreak of COVID-19 spreading worldwide has caused a decrease of available labor that affects the production of corn and soybeans leading to feed deficiencies. Therefore, it is important to find alternative sources of carbohydrates while safeguarding the health of animals [1–5]. Because fiber is considered to be an anti-nutritional factor, low-fiber diets were typically used when feeding non-ruminant livestock animals in the past [6]. However, fiber content positively correlates with the health of the animal and can regulate microbiota in mice and maintains the thickness of mucosa and decrease inflammatory-related cytokine production [7,8].

As a major by-product of wheat production, wheat bran (WB), comprises about 15% crude protein (CP), 28% carbohydrate, and 42% insoluble fiber with many anti-oxidation compounds included, such as ferulic acid, tocopherol, and lutein [9]. Therefore, WB has the potential to replace corn in poultry feed. However, there are also many phytate acids in WB that can decrease the nutrient utilization of a monogastric animal.

Among the restrictions of WB utilization, fermentation by probiotics and phytase addition can solve the problem of high phytate acids and fiber content. Cowieson et al [10] and Hamdi et al [11] indicated that the addition of phytase to feeds can increase the digestion of phosphorus, minerals, and amino acids of broilers and increase broilers’ bone strength. In addition, the research group of Attia et al [1–5] and Al-Harthi et al [1,3,5] reported that phytase could improve performance of broiler chickens regardless of phosphorus contents in broiler diet.

In recent years, the new concept of “postbiotic” has arisen. Unlike probiotic, prebiotic, or synbiotic, postbiotic emphasizes more on the metabolic changes due to probiotic in the fermentation matrix [12]. Because of the limited ability of probiotics, they may not be able to carry enzymes that degrade all anti-nutritional factors [13]. Therefore, the simultaneous addition of probiotics and exogenous enzymes may have a multiplying effect on the co-fermentation of WB. Phytates usually form a complex with fiber in the WB, and therefore cannot be degraded by phytase addition [10]. In the other hand, as reported by Chuang et al [14], *Saccharomyces cerevisiae* (*S. cerevisiae*) mainly secrets fiber-related enzymes, such as glucanase, mannanase, cellulase and xylanase, etc. Therefore, fermentation by *S. cerevisiae* can degrade fiber in WB, while it cannot decrease the phytase content in WB. Therefore, co-fermentation of WB by *S. cerevisiae* and phytase may be better than using *S. cerevisiae* only. Nevertheless, few studies have carried out co-fermentation of WB with probiotic and a specific enzyme. In theory, the postbiotic form from *S. cerevisiae* and phytase co-fermentation wheat bran (FWB) should increase the nutrient utilization and decrease the phytate content, and increase the growth performance of broilers. Furthermore, from the performance of *S. cerevisiae*, phytase and WB shown in previous studies, we predict that the FWB can increase the antioxidation function, enhance the tight junction (TJ) protein presentation, and decrease the inflammatory-related cytokine in broilers. We supposed that the use of FWB and WB to replace part of the feed will not have a negative impact on the growth performance of the broilers, and will improve their intestinal health. Therefore, we measured the broilers’ growth performance, intestinal morphology, microbiota composition, blood biochemical analysis, and mRNA expression in chicken peripheral blood mononuclear cells (chPBMCs).

## MATERIALS AND METHODS

### Saccharomyces cerevisiae and phytase co-fermented wheat bran preparation and characteristics

*Saccharomyces cerevisiae* and phytase co-FWB was created by the method as follows: Add 10,000 units phytase-6 and 100 mL 10^8^ colony-forming unit (CFU)/mL *S. cerevisiae* to 1 kg WB and let ferment at 30°C for 3 days. The FWB was moved from the incubator to a 50°C oven and staved for 1 day. The FWB was collected and stored at a 4°C refrigerator before use.

We analyzed the xylanase [15], protease [16], cellulase [17], and β-glucanase [17] activities in FWB by the methods mentioned above.

Extractable phosphorus measurement was modified from the previous study [18]. Briefly, add 1 gram of WB or FWB into 3 M HCl with 0.2% pepsin and soak it in 95°C water for 3 hours. After cooling, centrifuge it at 3,000 rpm for 10 minutes. Filter the supernatant through Advantec No.1 filter papers. Mix the filtrate and vanadium-ammonium molybdate solution. Then, centrifuge the filtrate at 6,000 rpm for 10 minutes, and measure the suspension of absorbance at 415 nm. The known concentration of potassium dihydrogen phosphate is used as the standard curve.

The methods of pentose and hexose measurement were modified from the phenol-sulfuric acid method. Briefly, add 1 mL 5% phenol solution and 5 mL sulfuric acid to FWB water extra. After incubation of 15 minutes, detect the absorbance at 480 nm for pentose and 490 nm for hexose. Take the xylose and glucose as the standard curve for pentose and hexose, respectively.

Add deionized water to FWB and stir it for 1 hour to measure the xylooligosaccharide. After stirring, centrifuge it at 3,000 rpm for 10 minutes and filter through Advantec No. 1 filter paper and 0.22 μm filter successively. Then, analyzed the sample by high performance liquid chromatography (HPLC) (HITACHI, Kyoto, Japan) equipped with a pump (L-2130), a column (TransgenomicCARBOSep CH0682 Pb, 300 mm×7.8 mm), a RI-detector (L-2490), an autosampler (L-2200) and a computer system with HPLC D-2000 Elite.

### Chicken peripheral blood mononuclear cells isolation

The methods of chPBMCs isolation were done according to Kaiser et al [19]. The whole blood of broilers (3 for each pen, 9 for each treatment) was collected and centrifuged at 200 g for 10 min to remove the supernatant. The blood cells, phosphate-buffered saline (PBS), and ficol were mixed gently and centrifuged at 200 g for 10 min. The chPBMCs were removed to new RNase free tube, and RPMI-1640 was added with 10% fetal bovine sera (FBS) (for cell test) or PBS (for quantitative polymerase chain reaction [qPCR]) and diluted to 10^7^ cells/mL.

### Nitric oxide assay of chicken peripheral blood mononuclear cells

chPBMCs were cultured in the RPMI-1640 and 10% FBS at 37°C and 5% CO_2_ for 2 hours. The 10 μL 1 ng/mL lipopolysaccharides (LPS) and 10 μL bacteria-free sample solution were added in the 10^7^ chPBMCs and co-incubated for 24 hours. After culturing, add 100 μL Griess reagent and detect the absorbance at 540 nm.

### 3-(4, 5-Dimethylthiazol-2-yl)-2,5-diphenyltetrazolium bromide (MTT) assay

chPBMCs were cultured in the RPMI-1640 and 10% FBS at 37°C and 5% CO_2_ for 2 hours. The 10 μL 1 ng/mL LPS and 10 μL bacteria-free sample solution were added in the 10^7^ chPBMCs and co-incubated for 48 hours. After co-incubating, adding 20 μL 0.5% 3-(4, 5-Dimethylthiazol-2-yl)-2,5-diphenyltetrazolium bromide (MTT) solution and culture for 4 h. After culturing, add 100 μL dimethyl sulfoxide and detect the absorbance at 570 nm.

### Animal experimental designs

The experiments were carried out at National Chung Hsing University, Taiwan, and all of the protocols followed those of the Animal Care and Use Committee (IACUC: 107-013) at October 2018. Animal experiment design methods followed by Teng et al [20] and were slightly modified. Briefly, 300 male one-day-old broilers (*Ross* 308) were divided into 5 groups: basal diet (control), 5% WB inclusion (5% WB), 5% FWB inclusion (5% FWB), 10% WB inclusion (10% WB), and 10% FWB inclusion (10% FWB). There were 20 broilers in each pen and 3 replicates for each treatment (each pen is about 3.24 m^2^, and at least 5 nipple drinkers each pen). The average initial body weight (49.0±0.5 grams/birds) was similar in each pen. A temperature-controlled house (33°C±0.5°C for one-day-old chicks and 21°C±1°C for chickens after 4 weeks old) was provided for this experiment during the whole rearing stage. In the whole rearing stage, there was 23-hour lighting and 1 hour dark per day, and free of additional medical treatment. Furthermore, feed and water were *ad libitum*. In order to standardise metabolic energy and CP in each treatment, the diet of each treatment group was recalculated to meet or exceed the nutrient requirements (NRC) [21] of broilers (Table 1). All of the proximate composition of FWB and each diet were measured according to the methods of AOAC [22]. The starter diet was offered for before 21 days old, and the finisher diet was offered for the broilers older than 22 days. Body weight, weight gain, and feed conversion rate (FCR) were measured at 21 and 35 days [8].

### Intestinal morphology

Thirty 35-day-old broilers (2 for each pen, 6 for each group totally) were used for the intestinal morphology test. The middle of the jejunum and the middle ileum of fasting one-day broilers were removed and fixed in formalin for 3 days. The samples were embedded in paraffin and stained with haematoxylin and eosin. The stained paraffin was sliced and observed under a light microscope, and a Motic Image Plus 2.0 analysis system (Motic Instruments, Richmond, Canada) was used to measure the villus height, crypt depth, and tunica muscular.

### Blood and serum characteristics

The blood of forty-five 35-day-old broilers (3 for each pen, 9 for each treatment) was collected for the blood and serum characteristic analysis. The 5 mL blood samples were stored at 4°C for 4 to 5 hours and centrifuged at 3,000 rpm for 10 minutes at 4°C to separate the blood cells and serum [8]. Blood cell and serum biochemical parameters were measured with a Automatic Biochemical Analyzer (Hitachi, 7150 auto-analyzer, Japan).

### Ash, calcium, and phosphorus contents of broiler femur

Thirty femurs (2 for each pen, 6 for each treatment) were taken from the 35-day-old broilers, and the ash content in a 600°C oven was measured. The calcium and phosphorus contents were measured by the methods described by AOAC [22]. Briefly, the ash of broilers’ femurs was collected and dissolved by hydrochloric acid and nitrite acid; 8 N potassium hydroxide and 2-Hydroxyl-1-(2-Hydroxyl-4-Sulful-1-Naphthylazo) were added to the solution and titrated by ethylenediaminetetraacetic acid for the calcium measure. The phosphorus measurement was modified from a previous study [18]. Here, the femurs’ ashes were dissolved in nitric acid and filtered through Advantec No.1 filter papers. The filtrate was added in the vanadium-ammonium molybdate solution, and the suspension absorbance at 415 nm was measured. The specific concentration of potassium dihydrogen phosphate was used for the standard curve.

### Microbial parameter in intestinal contents

Thirty 35-day-old broilers (2 for each pen, 6 for each treatment) were used for the intestinal microbial content measurement. The chyme in the ileum and the chyme in the cecum were squeezed out and diluted by sterilized PBS and cultured by the De Man, Rogosa, and Sharpe agar (Difco Lactobacilli MRS Agar) for *Lactobacillus* spp. and by tryptose sulfite cycloserine agar (GranuCult TSCagar, Merck, Darmstadt, Germany) for *Clostridium perfringens* at 37°C and anaerobic incubator for 48 hours. The CFU on the agars was counted after culturing.

### Chicken peripheral blood mononuclear cells’ and intestine cells’ total RNA isolation and qPCR

Fifty micrograms of ileum of 35-day-old broilers (2 for each pen, 6 for each treatment) were removed and soaked in 1 mL RNAzol (Molecular Research Center, Inc, Cincinnati, OH, USA). The ileum tissue was homogenous and stored in a −20°C refrigerator before being used.

The methods of mRNA isolation were done according to the manufacturer’s protocol of SuperScript FirstStrand Synthesis System reagent (Invitrogen, Woburn, MA, USA). 2× SYBR GREEN PCR Master Mix-ROX (Gunster Biotech, Co., Ltd., Foster City, CA, USA), cDNA, deionized water, and each primer were mixed at the ratio of 5:1.2:1.8:1. StepOnePlus Real-Time PCR System (Thermo Fisher, Waltham, MA, USA) was used to detect qRT-PCR performance. The 2^−ΔΔCt^ method was used to calculate the relative mRNA expression level, and β-actin was used as the housekeeping gene for normalization. Gene-specific primers were utilized according to the genes of Gallus gallus (chicken) and according to Genbank given as Supplementary Table S1.

### Statistical analysis

The data collected were statistically analyzed using general linear models procedure of SAS software (SAS 9.4, 2018) following a completely randomized design. Data on the dietary treatments were subjected to analysis of variance using Statistical Analysis System Institute Package (SAS) and the mean values were compared using Tukey test with a significant level at p<0.05.

The mathematic model was:

$${\text{Y}}_{\text{ij}}=\mu +{\text{D}}_{\text{i}}+{ɛ}_{\text{ij}}$$

Where Y_ij_ is the measurement on average of birds in pen j, dietary treatment i; μ is the overall mean; D_i_ is the fixed effect of dietary treatment i; ɛ_ij_ is the residual term that ɛ_ij_∩N (0, σ^2^ɛ). The experimental units were different depend on the experiments, including *in vitro* test (WB and FWB product or well of chPBMCs culturing plate), and *in vivo* test (per chicken).

## RESULTS

### Wheat bran and fermented wheat bran characteristics

There were no xylanase, protease, cellulase, and β-glucanase in WB, but after fermentation, the enzymes mentioned above increased to 153, 549, 290, and 147 U/g DM, respectively (Supplementary Table S2). WB has little soluble pentose and hexose; however, after fermentation, pentose, and hexose respectively increased from 75.3 to 194 and 127 to 298 mg/g DM (Supplementary Table S2). After fermentation, the FWB CP showed an increase from 17.8% to 20.3% DM, and the hemicellulose content also increased from 26.8% to 30.1% DM compared to WB (Supplementary Table S2). Wheat bran has17.8 μmol/g DM extractable inorganic phosphorus; after being fermented by *S. cerevisiae*, phytase, and *S. cerevisiae* with phytase, the extractable inorganic phosphorus increased to 79.2, 198, and 337 μmol/g DM (Supplementary Figure S1).

With 1 ng LPS, the nitric oxide (NO) production in creased to about 120 μmol on 10^7^ chPBMCs. However, both 5% FWB and 10% FWB additions can significantly decrease (p<0.05) the NO production of 10^7^ chPBMCs, which were stimulated by 1 ng LPS (Supplementary Figure S2). Furthermore, in the MTT assay, 10% FWB can increase the survival rate (p<0.05) of LPS to stimulate chPBMCs (Supplementary Figure S3).

### Animal performances

In the starter stage (1 to 21 d), the 10% FWB group had the best WG (p<0.05), and the 5% FWB and 10% FWB groups had the lowest FCR in data (1.06 and 1.07, respectively) (Table 2). There were no significant differences in body weight, feed consumption, and FCR in the finisher stage (22 to 35 d), but in the whole stage (1 to 35 d) the 5% and 10% FWB groups had the best FCR (both of them were 1.52, p<0.05) (Table 2).

Villus height increased in the 10% WB and 10% FWB groups (1,356 and 1,396 μm, respectively, and the control group was only 1,201 μm, p<0.05). However, there were no significant differences in crypt depth, tunica muscularis, and the villus:crypt ratio in the jejunum. In the ileum, there were no significant differences in villus height, crypt depth, and tunica muscularis; however, the 5% FWB group had the highest villus:crypt ratio (6.35 compared to 5.65 in the control group) (Table 3). Supplementary Figure S4 shows the photomicrography of jejunum and ileum of 35-day-old broilers.

From the data in Table 4, 5% FWB and 10% WB groups had an increased red blood cell concentration in blood (p< 0.05), and the 5% FWB group had the highest hemoglobin content (8.53 g/dL, compared to 7.83 of the control group, p<0.05). Furthermore, the glucose in blood increased from 201 to 237, 247, 255, and 276 mg/dL respectively in the control, 5% WB, 5% FWB, 10% WB, and 10% FWB groups (p< 0.001). Both uric acid (UA) and blood urea nitrogen (BUN) decreased in the treatment groups compared to the control group (p<0.05), and there were no significant differences in the amount of inclusion and for fermentation or not.

From the data in Table 4, the 5% FWB and 10% FWB groups both had an increased ash content in the femurs (39.9% and 45.5% DM, respectively, compared to 35.5% DM in the control group, p<0.05), and there was a little dose effect. The calcium content also increased from 10.3% to 11.6% and 13.0% DM in the 5% FWB and 10% FWB groups compared to the control group in data (p>0.05).

### Microbe parameter

There were no significant differences in the microbial parameter in the ileum but in the caecum the numbers of *Lactobacillus* spp. had a significant increase in the 10% WB and 10% FWB groups. Although there were no significant differences in the number of caecum *Clostridium perfringens*, it was positively correlated with the interferon-gamma mRNA expression in the chPBMCs test (Table 3).

### mRNA expression

**Figure 1:**
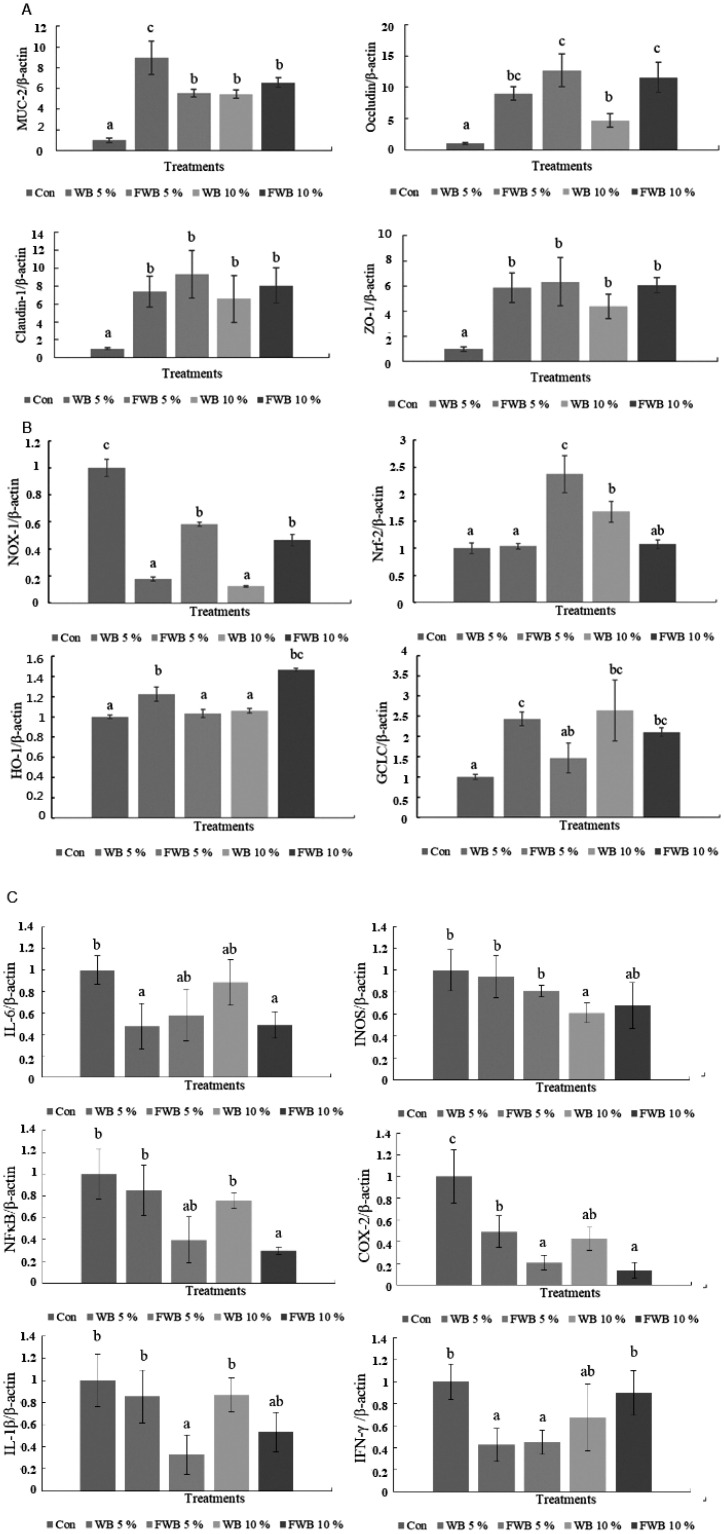
The mRNA expression level. (A) Tight junction genes in chicken ileum at 35 d; (B) antioxidant genes in chPBMCs at 35 d; (C) immunomodulatory genes in chPBMCs at 35 d. chPBMCs, chicken peripheral blood mononuclear cells. ^a–c^ Means within the same rows without the same superscript letter are significantly different (p<0.05).

In the ileum, the mucin 2 (MUC2) mRNA expression increased in all treatment groups compared to the control group (p<0.05). The ileum mRNA expression of TJ occludin, claudin-1, and zonula occludens-1 (ZO-1) are also shown in Figure 1A. The data showed that all of the treatment groups had increased TJ mRNA expression compared to the control group (p<0.05). Among them, 5% FWB and 10% FWB could increase the occludin mRNA expression, especially compared to the unfermented WB group.

In the expression of anti-oxidation-related mRNA on chPBMCs, nuclear factor erythroid 2-related factor 2 (Nrf-2) increased significantly in the 5% FWB group (p<0.05), and the 5% WB and 10% FWB groups increased significantly on the heme oxygenase-1 (HO-1) mRNA expression (p<0.05). Nicotinamide adenine dinucleotide phosphate-oxidase-1 (NOX-1) and glutamate-cysteine ligase catalytic (GCLC) have complementary results in all treatments. The lower NOX-1 and higher GCLC mRNA expressions were in the 5% WB and 10% WB groups compared to the control group (p<0.05) (Figure 1B).

The data of pro-inflammatory cytokines, such as interleu kin-6 (IL-6), induciblenitric oxide synthases (iNOS), nuclear factor-κB (NF-κB), IL-1β, cyclooxygenase-2 (COX-2), and interferon-gamma (IFN-γ), are shown in Figure 1C. The NF-κB and IL-1β mRNA expressions in chPBMCs significantly decreased in the 5% FWB and 10% FWB groups compared to the control group (p<0.05), but there was no dose effect. Furthermore, COX-2 mRNA expression in chPBMCs had a similar effect in the 5% FWB and 10% FWB groups, and the 5% WB and 10% WB groups also had a significant decrease compared to the control group; the 5% WB and 5% FWB groups had the lowest IFN-γ mRNA expressions. There were no significant differences in the iNOS mRNA expression of each group.

## DISCUSSION

Previous studies pointed out that fiber can improve the intestinal health of animals [8]. Fibers can be used as prebiotics for probiotic fermentation and promote probiotics to produce a lot of functional substances [13]. However, natural botanical raw materials are often accompanied by complex fiber composition as well as fiber-encapsulated minerals, phenolic acids, inorganic phosphorus, phytic acid, and other nutrients [11]. Coupled with the limited time of fiber passage through the intestine, it is difficult for normal intestinal flora to completely digest the fibers [23]. Therefore, the feasibility of directly utilizing high-content fibers in a diet has been evaluated. In recent years, in addition to emerging concepts other than probiotics, prebiotics, and synbiotics, postbiotic has been defined as a matrix of probiotics fermented prebiotics *in vitro*, containing a variety of substrates, probiotics, and microbes secretion by the fermentation [13]. Many enzymes will be produced during fermentation, which can promote intestinal health and nutrient digestion and absorption of the host [24,25]. In addition, these enzymes can also produce more oligosaccharides and simple sugars when fermenting WB [26], including D-xylose, xylobiose, and xylotriose, and the oligosaccharides which can improve villus growth and immunity [27].

Because the phytic acid in WB is present in complex fibers [4], and *S. cerevisiae* could only produce fiber or protein-related enzymes such as cellulase, xylanase, protease, and glucanase but not phytase. Therefore, fermentation WB by *S. cerevisiae* is less helpful for the release of inorganic phosphorus. However, *S. cerevisiae* fermentation can increase the phytic acid release and increase the function of phytase. Therefore, co-fermentation with *S. cerevisiae* and phytase will increase the release of inorganic phosphorus.

Because of the fermentation, FWB has more simple sugars, oligosaccharide, enzyme, and inorganic phosphorus content than WB, and these can increase the nutrient absorption of broilers [25]. Furthermore, high fiber content is positively related to intestinal health and will also make the microbiota stable [8]. In the early stage of animal growth, the composition of intestinal flora is important, because microbiota will control the growth and health of animals [8]. The 10% FWB group had higher body weight gain in the starter stage (1 to 21 d) of broilers, and the lower FCR in diets can be seen in the 5% FWB and 10% FWB groups. The results mentioned above might be because the fiber in the 10% FWB group can stabilize the microbiota better than the 5% FWB group and contains more functional metabolites, as well as phytase, to promote the health of broilers [28,29]. Teng et al [20] indicate that *Bacillus amyloliquefaciens*, and *S. cerevisiae* fermented WB inclusion will not increase broilers’ growth performances. Nevertheless, Santos et al [28] indicated that the addition of phytase (500 phytase unit [FTU]/kg) in broilers’ diet can increase body weight gain in the starter stage, but does not affect the finisher stage. This may be due to gut maturation and less inorganic phosphorous requirements during the finisher period as well as stability of gut microbiota [2–5]. The result mentioned above confirmed that phytase is one of the necessary elements of FWB in the broilers’ growth at the starter stage. In the finisher stage, the positive effect of the 10% FWB group decreases, but increases in the 5% FWB group. Although the FCR of finisher broilers is still lower in the 5% FWB and 10% FWB groups than in the other groups, there is no significant effect on weight gain.

From the above data, it can be inferred that the use of 10% FWB in the early stage and the use of 5% FWB in the finisher stage may be better for the body weight gain of broilers. Furthermore, FWB has a better effect than WB inclusion.

As for the higher fiber treatment, 10% WB and 10% FWB significantly increased the number of *Lactobacillus* spp. in the cecum of broilers. In a previous study, fiber addition to a diet has a positive effect on *Lactobacillus* spp. growth [28]. In addition, in current research, fiber content was positively related to villus height in the jejunum, but had no significant effect on the ileum. However, the best villus:crypt ratio appeared in the 5% FWB group, and the villus:crypt ratio is positively correlated with the absorption ability of nutrients by animals. This result also echoes the growth performance, because the 5% FWB group had a better effect in the finisher stage than in the starter period. The high-fiber diet is also associated with the growth of villi, while also stabilizing the thickness of intestinal mucosa and maintaining the impermeability of the mucosa [8]. Although 0.3% sugar beet pulp (SBP) or rice hull (RH) addition cannot increase broilers’ villus height, adding 0.3% SBP or RH together with 0.1% organic acid had a slightly increased effect on jejunum villus height [28].

Similar results can also be seen in the blood biochemical analysis. The hemoglobin (Hb) content is related to the oxygen-carrying capacity of the animal and is positively correlated with the efficiency of nutrient utilization. Therefore, the 5% FWB group has better results on improving nutrient utilization at the 35-day-old. In addition, elevated blood sugar levels are associated with fiber content and fermentation. Both the reduction of BUN and UA in the blood are related to the utilization of protein [30]. Fiber intake also increases the antioxidant activity of broilers, which may enhance the liver’s detoxification function to amines and the function of the kidney to filter urea nitrogen, resulting in decreased levels of UA and BUN in the blood [31]. The high concentration of BUN in the blood indicates an abnormality in renal function [31]. In poultry, urease can decompose UA to form BUN and finally re-hydrolyze BUN to amine and carbon dioxide [31]. Therefore, low levels of UA in the blood may also be one of the reasons for the low BUN content.

As an important component supporting the body of the animal, the skeleton is mainly composed of minerals such as calcium and phosphorus and organic substances such as proteins [12]. FWB can significantly increase the ash content in the bone of broilers and is positively correlated with the amount of FWB inclusion. As previous studies indicated, phytase addition (1,000 FTU/kg diet) in the diet can promote bone strength and ash content in bone [12], and we present similar results. In the phosphorus-calcium ratio (P/Ca ratio), there is a tendency to decrease in both of the replace amount and fermentation WB. Lower P/Ca ratios denote higher bone strength and can reduce the incidence of chicken osteoporosis. Therefore, the data show that the FWB treatment group has a positive impact on bone health, especially in the 10% FWB treatment group.

The poultry industry often encounters climatic or environmental challenges. One of the common problems is the response to oxidative stress, inflammatory response, and intestinal epithelial damage of poultry [32]. The mucosal layer above the intestinal epithelial cells can effectively isolate intestinal microbes and intestinal epithelial cells, but it is easily damaged by a low-fiber diet, further leading to increased susceptibility of intestinal epithelial cells [8]. In addition, the size of the gap between intestinal epithelial cells is mainly controlled by TJ-related proteins, including occludin, claudin, and zonula occludens (ZO) families [33]. Therefore, MUC2, a major protein in the mucosa, shows a significant increase in mRNA expression, thereby increasing the distance between the microbes and the intestinal epithelial cells [8]. Claudins and occludin are transmembrane proteins that are responsible for regulating the size of the intercellular space [33]. Two adjacent epithelial cells can be “stacked” together by the combination of claudins and occluding [33]. ZO-1 is present in intestinal epithelial cells and can be attached to claudins and occludin to increase the stability of TJ [33].

From the results of this report, the TJ-related mRNA ex pression exhibits a significant increase, indicating that both WB and FWB can significantly improve the performance of TJ. Furthermore, the effects of FWB treatment on occludin mRNA expression are higher than WB treatment. The results may be due to short-chain fatty acids (SCFAs) produced by *S. cerevisiae* being able to promote the health of intestinal epithelial cells and the performance of TJ protein [34].

The health of intestinal epithelial cells is also related to the expression of inflammatory factors in animals, and the role of inflammation is not only related to immune regulation, but also the decreased oxidation stress of animals. Therefore, the health of intestinal epithelial cells is closely related to the regulation of antioxidant capacity and the regulation of immunity in the blood of animals [35,36]. The Nrf-2 is an important upstream regulator of antioxidants and normally binds to kelch-like ECH-associated protein 1 (Keap-1) in the cytoplasm, and will be activated and separated with Keap-1 by the stimuli such as pathogen and oxidative pressure [35,36]. After activation, Nrf-2 binds to Maf and reacts with an antioxidant responsive element to promote downstream antioxidant gene expression, including GCLC and HO-1 [37]. GCLC is a catalytically active site of glutamate cysteine ligase (GCL), which can synthesize GSH and neutralize free radicals [35,36]. HO-1 acts as a metabolic rate-limiting enzyme for heme, which cleaves heme into biliverdin (BV) and reduces it to bilirubin through the naturally occurring BV reductase in the cytoplasm [37]. The levels of BV and bilirubin in the blood can neutralize excess hydrogen peroxide [37]. The NOX-1 is a member of the pro-oxidase group, which can promote the production of reactive oxygen species (ROS) [36]. The ROS is like a double-edged sword, as it not only can destroy the pathogen but can also damage the host cell [36]. However, in normal feeding environments, since the broilers are not infected, it is not necessary to activate NOX-1 to produce ROS. Here, 5% FWB and 10% FWB can significantly increase the Nrf-2 mRNA expression. Compared to the control group, all the treatments have a higher tendency of GCLC mRNA expression, which can protect broilers from sudden oxidative stress. The NOX-1 data prove that WB and FWB are non-toxic feeds and do not cause additional oxidative stress.

In addition to oxidative stress, another protective mechanism in animals is their inflammatory response. An inflammatory response protects the host from pathogens, but it can also cause apoptosis in the host due to excessive inflammatory reactions [38]. The cell wall of *S. cerevisiae* is mainly composed of β-glucan and mannan, both of which have the function of encapsulating toxins and can reduce the damage caused by LPS to cells [39], and these function can decrease the stimuli from LPS and decrease the NO release from chPBMCs.

The main regulators of inflammation are NF-κB and in terleukin family [38]. NF-κB is present as inhibitor kappa B (IκB) in the cytoplasm and is not active in a normal situation, but when stimuli exist, IκB kinase will be activated and can spur IκB to become NF-κB [38] and it can activate iNOS, and COX-2, and positive feedback on IL-1β [40]. As one of the most important members of the IL family, IL-1β is upstream of NF-κB. However, IL-1β is inhibited by β-glucan [40], and a fiber-rich diet will also decrease the inflammatory-related cytokine release compared to a fiber-free diet [8]. iNOS can be activated by NF-κB and promotes the production of NO by cells [40]. As an RNS, NO has a similar function to ROS, which can cause the death of pathogens and the accumulation of immune cells when infected [35].

When an animal is traumatized or infected, it causes a large secretion of COX-2, promotes the conversion of arachidonic acid to prostaglandin E2 and prostaglandin F2α, induces macrophage aggregation, and induces an inflammatory response [40]. The IFN-γ is also associated with infection and promotes NF-κB activation and ROS production in response to external stress when the host is infected [38]. Both WB and FWB inclusion have a decreasing trend for IL-1β, and the trend is the same as that of NF-κB, especially in FWB inclusion. Plant fiber has the effect of inhibiting IL-1β [35], and FWB contains a lot of *S. cerevisiae* cell walls, which can also inhibit IL-1β function. In addition, WB and FWB will also affect intestinal microbiota can lead to different inflammatory responses in the host. 10% WB and 10% FWB inclusion will significantly increase the *Lactobacillus* spp. number in the caecum, and Hegazy and El-Bedewy [38] indicated that the addition of 10^10^ CFU of *Lactobacillus delbruekii* and *L. fermentum* can decrease the IL-6 and NF-κB mRNA expressions.

The above results show that both FWB and WB can achieve an anti-inflammatory effect by inhibiting the expression of IL-1β, wherein the effect of FWB is better than that of WB, but the amount of substitution has little effect on the degree of inclusion.

## CONCLUSION

Based on the above results, a high WB diet does increase the health of animals. However, although both WB and FWB promote the performance of TJ mRNA expression and improve the antioxidant capacity of broilers, FWB is better in decreasing inflammatory response. In addition, data also showed that, 10% FWB inclusion show better growth performance than other treatments in starter stage but finisher stage of broilers. Therefore, it is confirmed that, as a postbiotic, there was higher nutrition value in FWB than WB and worthy to be a feed inclusion strategy.

## Supplementary Information



## Figures and Tables

**Table 1:** Composition and calculated analysis (g/kg as fed) of the basal and experimental diet for broilers

Items	Con	5% WB	5% FWB	10% WB	10% FWB
**Starter diet (1 to 21 days, g/kg)**					
Yellow corn	523	431	432	406	404
Soybean meal (CP 44.0%)	323	197	193	139	136
Full fat soybean meal	53.0	223	226	257	262
Soybean oil	30.0	30.0	30.0	30.0	30.0
Fish meal (CP 65.0%)	30.0	30.0	30.0	30.0	30.0
Monocalcium phosphate	14.0	13.0	13.0	12.0	12.0
Calcium carbonate	16.0	16.0	16.0	16.0	16.0
NaCl	3.40	3.30	3.30	3.20	3.20
DL-Methionine	3.50	3.40	3.40	3.40	3.40
L-Lysine-HCl	1.90	1.60	1.60	1.60	1.6
Choline-Cl	0.80	0.80	0.80	0.80	0.8
Vitamin premix^1)^	1.00	1.00	1.00	1.00	1.00
Mineral premix^2)^	1.00	1.00	1.00	1.00	1.00
Wheat bran	0	50.0	0	100	0
Fermented wheat bran	0	0	50.0	0	100
Total	1000	1000	1000	1000	1000
Calculated nutrient value					
DM (%)	88.1	88.7	88.7	88.7	88.7
CP (% DM)	23.1	23.0	23.0	23.1	23.0
Calcium (% DM)	1.05	1.05	1.05	1.05	1.05
Total phosphorus (% DM)	0.720	0.800	0.800	0.760	0.760
Available phosphorus (% DM)	0.500	0.500	0.500	0.500	0.500
Methionine+cystein (% DM)	0.730	0.720	0.720	0.720	0.720
Lysine (% DM)	1.22	1.21	1.20	1.22	1.19
ME (kcal/kg DM)	3050	3050	3050	3050	3050
Chemical analysis value					
DM (%)	88.0	88.5	88.6	88.7	89.0
CP (% DM)	23.0	23.1	23.0	23.0	23.0
Crude fat (%)	6.72	9.54	9.33	9.87	9.76
**Finisher diet (22 to 35 days, g/kg)**					
Yellow corn	564	501	500	448	447
Soybean meal (CP 44.0%)	275	52.0	49.0	89.0	86.0
Full fat soybean meal	60.0	325	328	263	266
Soybean oil	41.0	13.0	13.0	40.0	41.0
Fish meal (CP 65.0 %)	25.0	25.0	25.0	25.0	25.0
Monocalcium phosphate	13.0	13.0	13.0	14.0	14.0
Calcium carbonate	12.0	12.0	12.0	11.0	11.0
NaCl	2.90	2.70	2.70	2.80	2.80
DL-Methionine	1.30	0.70	0.70	0.90	0.90
L-Lysine-HCl	3.50	3.20	3.20	3.30	3.30
Choline-Cl	0.80	0.8	0.80	0.80	0.80
Vitamin premix^1)^	1.00	1.00	1.00	1.00	1.00
Mineral premix^2)^	1.00	1.00	1.00	1.00	1.00
Wheat bran	0	50.0	0	100	0
Fermented wheat bran	0	0	50.0	0	100
Total	1,000	1,000	1,000	1,000	1,000
Calculated nutrient value					
DM (%)	88.2	88.5	88.7	88.8	89.0
CP (% DM)	21.0	21.1	21.0	21.0	21.1
Calcium (% DM)	0.901	0.902	0.901	0.901	0.902
Total phosphorus (% DM)	0.661	0.742	0.742	0.704	0.703
Available phosphorus (% DM)	0.451	0.452	0.450	0.453	0.452
Methionine+cysteine (% DM)	0.960	0.960	0.960	0.960	0.960
Lysine (% DM)	1.01	1.01	1.00	1.01	1.02
ME (kcal/kg DM)	3,175	3,175	3,175	3,175	3,175
Chemical analysis value					
DM (%)	88.0	88.4	88.6	88.7	89.03
CP (% DM)	21.0	21.0	21.0	21.0	21.0
Crude fat (%)	8.01	10.92	10.88	11.1	11.1

CP, crude protein; DM, dry matter; ME, metabolic energy.

1) Vitamin (premix content per kg diet): vit. A, 15,000 IU; vit. D_3_, 3,000 IU; vit. E, 30 mg; vit. K_3_, 4 mg; thiamine, 3 mg; riboflavin, 8 mg; pyridoxine, 5 mg; vitamin B_12_, 25 μg; Ca-pantothenate, 19 mg; niacin, 50 mg; folic acid, 1.5 mg; and biotin, 60 μg.

2) Mineral (premix content per kg diet): Co (CoCO_3_), 0.255 mg; Cu (CuSO_4_ 5H_2_O), 10.8 mg; Fe (FeSO_4_ H_2_O), 90 mg; Mn (MnSO_4_ H_2_O), 90 mg; Zn (ZnO), 68.4 mg; Se (Na_2_SeO_3_), 0.18 mg.

**Table 2:** Effect of wheat bran or *Saccharomyces cerevisiae* fermented wheat bran supplemention on growth performance of 1 to 35 d-old broilers

Items	Treatments^1)^					SEM	p-value

	Con	5% WB	5% FWB	10% WB	10% FWB		
1 to 21 d							
Body weight (g/bird)	797^b^	729^b^	762^b^	783^b^	873^a^	21.0	0.0078
Weight gain (g/bird)	748^b^	680^b^	713^b^	734^b^	824^a^	21.0	0.0075
Feed consumption (g/bird)	959	889	810	980	934	55.3	0.274
FCR	1.20	1.21	1.06	1.25	1.07	0.051	0.0788
22 to 35 d							
Body weight (g/bird)	2,131	1,959	2,078	2,054	2,075	33.8	0.0507
Weight gain (g/bird)	1,333	1,230	1,316	1,271	1,202	32.4	0.0763
Feed consumption (g/bird)	2,518	2,334	2,342	2,325	2,225	82.0	0.235
FCR	1.89	1.89	1.78	1.83	1.85	0.052	0.529
1 to 35 d							
Weight gain (g/bird)	2,082	1,910	2,029	2,005	2,027	33.8	0.0504
Feed consumption (g/bird)	3,477^a^	3,222^b^	3,152^b^	3,305^b^	3,159^b^	47.2	0.0035
FCR	1.63^a^	1.65^a^	1.52^b^	1.61^a^	1.52^b^	0.021	0.0022

SEM, standard error of the mean; FCR, feed conversion rate.

1) WB, wheat bran; FWB, fermented wheat bran.

a,b Means within the same rows without the same superscript letter are significantly different (p<0.05).

**Table 3:** Effect of wheat bran or *Saccharomyces cerevisiae* fermented wheat bran supplemention on intestinal morphology and microbiota of 35 d-old broilers

Items	Treatments^1)^					SEM	p-value

	Con	5% WB	5% FWB	10% WB	10% FWB		
Intestinal morphology							
Jejunum							
Villus height (μm)	1,202^c^	1,286^bc^	1,176^c^	1,356^b^	1,397^ab^	58.5	0.0050
Crypt depth (μm)	221	209	170	198	202	15.9	0.271
Tunica muscularis (μm)	215	184	200	208	209	19.4	0.816
Villus:crypt	5.74	6.17	6.98	7.64	7.10	0.52	0.116
Ileum							
Villus height (μm)	958	958	1,036	966	1,017	64.7	0.859
Crypt depth (μm)	183	169	164	206	218	16.6	0.0514
Tunica muscularis (μm)	235	260	220	237	249	24.8	0.830
Villus:crypt	5.65^ab^	5.76^ab^	6.35^a^	4.73^b^	4.97^b^	0.35	0.0055
Microbial parameter (log CFU/g)							
Ileum							
*Clostridium perfringens*	7.20	7.95	7.79	7.82	7.81	0.320	0.528
*Lactobacillus* spp.	8.93	8.45	8.67	8.54	7.17	0.418	0.094
Caecum							
*Clostridium perfringens*	8.00	7.40	7.22	8.23	9.39	0.472	0.058
*Lactobacillus* spp.	8.58^b^	9.24^ab^	9.25^ab^	10.05^a^	9.97^a^	0.309	0.039

SEM, standard error of the mean; CFU, colony-forming unit.

1) WB, wheat bran; FWB, fermented wheat bran.

a–c Means within the same rows without the same superscript letter are significantly different (p<0.05).

**Table 4:** Effect of wheat bran or *Saccharomyces cerevisiae* fermented wheat bran supplemention on serum and femurs characteristics of broilers (35 d)

Items	Treatments^1)^					SEM	p-value

	Con	5% WB	5% FWB	10% WB	10% FWB		
Serum							
RBC (10^6^/μL)	2.28^ab^	2.19^b^	2.42^a^	2.43^a^	2.28^ab^	0.064	0.0445
Hb (g/dL)	7.83^bc^	7.54^c^	8.53^a^	8.23^ab^	8.07^abc^	0.198	0.0105
SGOT (U/L)	211	182	194	198	187	8.50	0.174
SGPT (U/L)	2.14	1.77	2.12	1.48	1.44	0.460	0.726
GLU (mg/dL)	201^c^	237^b^	247^ab^	255^ab^	276^a^	10.8	<0.001
BUN (mg/dL)	1.67^a^	1.22^b^	1.11^b^	1.00^b^	1.00^b^	0.153	0.0209
UA (mg/dL)	4.77^a^	3.66^b^	3.26^b^	3.31^b^	3.18^b^	0.356	0.0166
CHOL (mg/dL)	110	104	113	112	111	5.01	0.802
TG (mg/dL)	71.2	83.3	83.4	88.1	76.0	7.40	0.505
HDL-C (mg/dL)	77.0	73.1	78.3	78.1	77.2	3.50	0.870
Femurs							
Ash (% DM)	35.5^b^	31.8^b^	39.9^ab^	35.1^b^	45.5^a^	3.14	0.0473
P content (% DM)	7.93^ab^	6.60^c^	7.78^bc^	6.82^a^	8.06^bc^	1.01	0.0030
Ca content (% DM)	10.3	9.4	11.6	10.7	13.0	1.48	0.459
P/Ca ratio	0.77^a^	0.70^ab^	0.67^bc^	0.64^bc^	0.62^c^	0.04	0.0251

SEM, standard error of the mean; RBC, red blood cell; Hb, hemoglobin; GLU, glucose; BUN, blood urea nitrogen; UA, uric acid; SGOT, serum glutamic oxaloacetic transaminase; SGPT, serum glutamic pyruvic transaminase; CHOL, cholesterol; TG, triglycerides; HDL, cholesterol-high-density lipoprotein; DM, dry matter.

1) WB, wheat bran; FWB, fermented wheat bran.

a–c Means within the same rows without the same superscript letter are significantly different (p<0.05).
