# Supplementary material for: Effects of Saccharomyces cerevisiae and phytase co-fermentation of wheat bran on growth, antioxidation, immunity and intestinal morphology in broilers
Source: Anim Biosci. 2020 Oct 13;34(7):1157–68. doi: 10.5713/ajas.20.0399 (PMC8255880; doi:10.5713/ajas.20.0399)
Supplement: Supplementary file 1 [file ajas-20-0399-suppl.pdf]

Electronic Supplements

Supplements figure captions

Supplement figure 1. The extractable phosphorus (μmol/g DM) of wheat bran (WB) with different treatments. Wheat bran (WB), wheat branfermented by *Saccharomyces cerevisiae* without phytase (SC), 500 FTU phytase per kilogram wheat bran (WB+phytase) and 500 FTU phytase per kilogram *Saccharomyces cerevisiae* fermented wheat bran (FWB). ^{a,b}Means within the same rows without the same superscript letter are significantly different ($P < 0.05$).

Supplement figure 2. Nitric oxide release amount of phosphate buffer solution (PBS, control), 1 ng/ml lipopolysaccharide (LPBS, negative control), 50 mg/mL FWB extraction and 1 ng/ml LPS included (50 mg/ml LFWB) and 100 mg/mL FWB extraction and 1 ng/ml LPS included (100 mg/ml LFWB). ^{a,b}Means within the same rows without the same superscript letter are significantly different ($P < 0.05$).

Supplement figure 3. MTT assay of phosphate buffer solution (PBS, control), PBS and 1 ng/ml lipopolysaccharide (LPBS, negative control), 50 mg/mL FWB extraction and 1 ng/ml LPS included (50 mg/ml LFWB) and 100 mg/mL FWB extraction and 1 ng/ml LPS included (100 mg/ml LFWB). ^{a,b}Means within the same rows without the same superscript letter are significantly different ($P < 0.05$).

Supplement figure 4. Photomicrography of jejunum and ileum of 35-day-old broilers fed with control, WB and FWB. (A-E) jejunum, respectively control, 5% WB, 5% FWB, 10% WB, and 10% FWB. (F-J) ileum, respectively control, 5% WB, 5% FWB, 10% WB, and 10% FWB. Haematoxylin and eosin stain. 40╳.

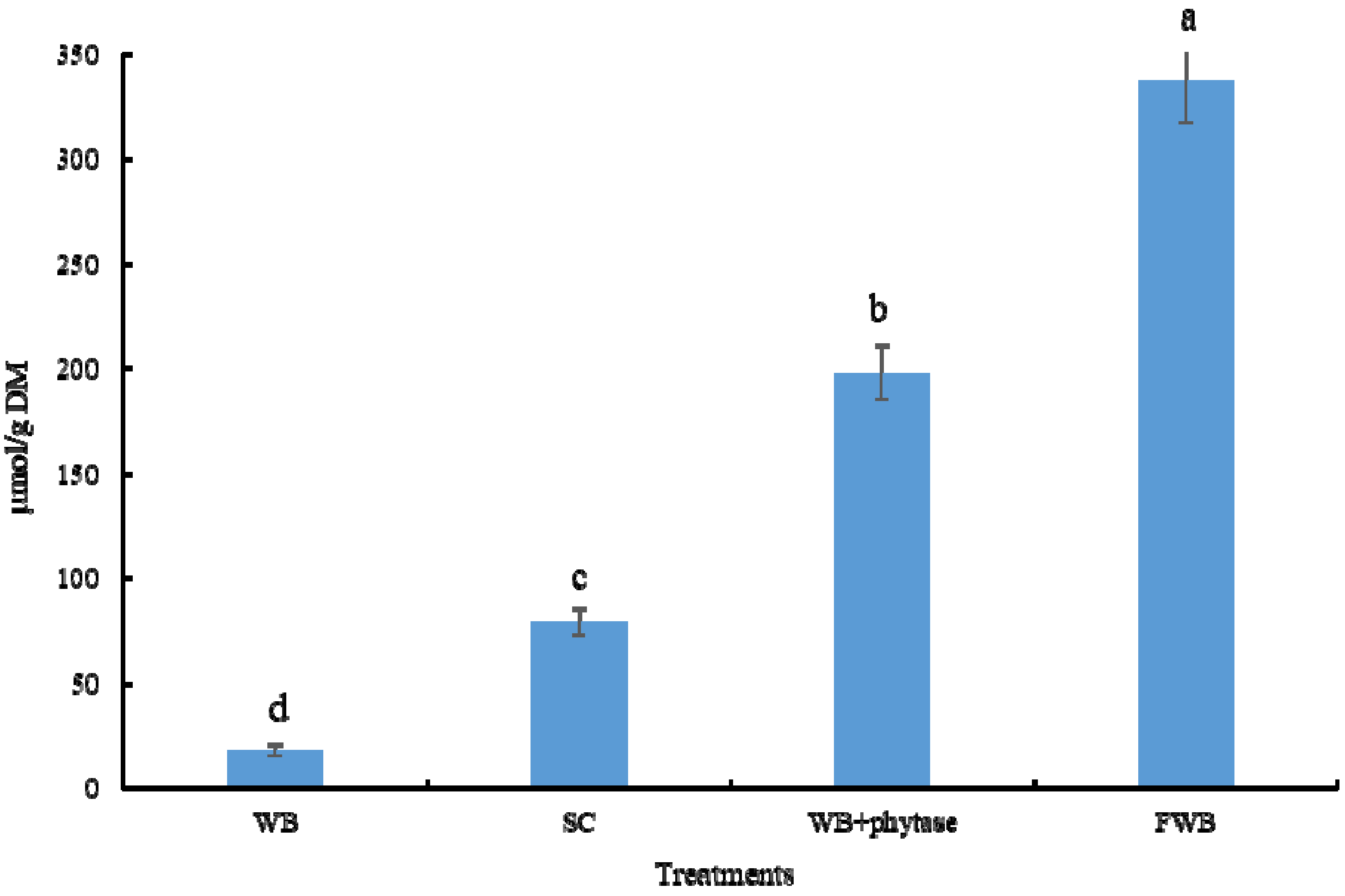

Supplement figure 1

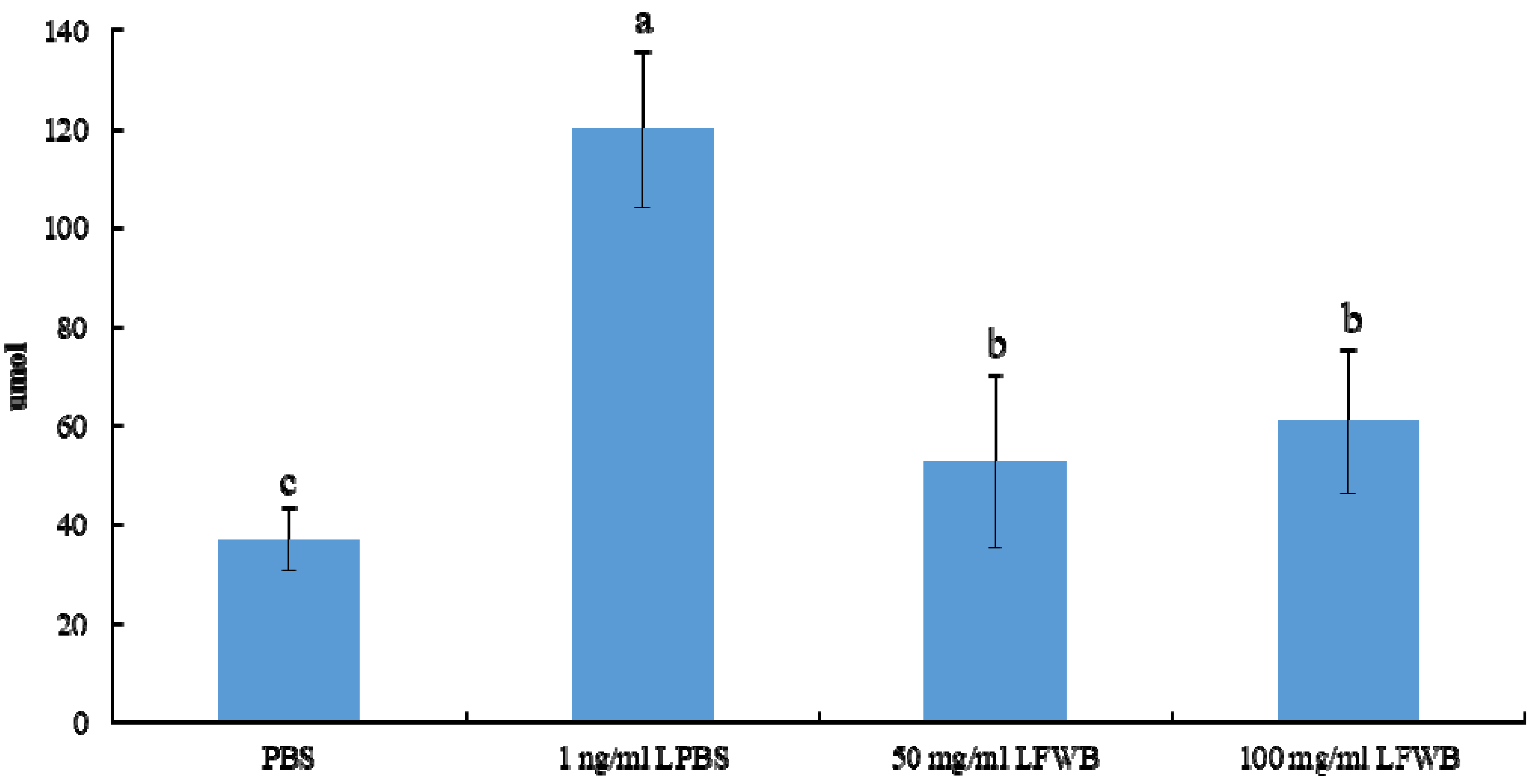

Supplement figure 2

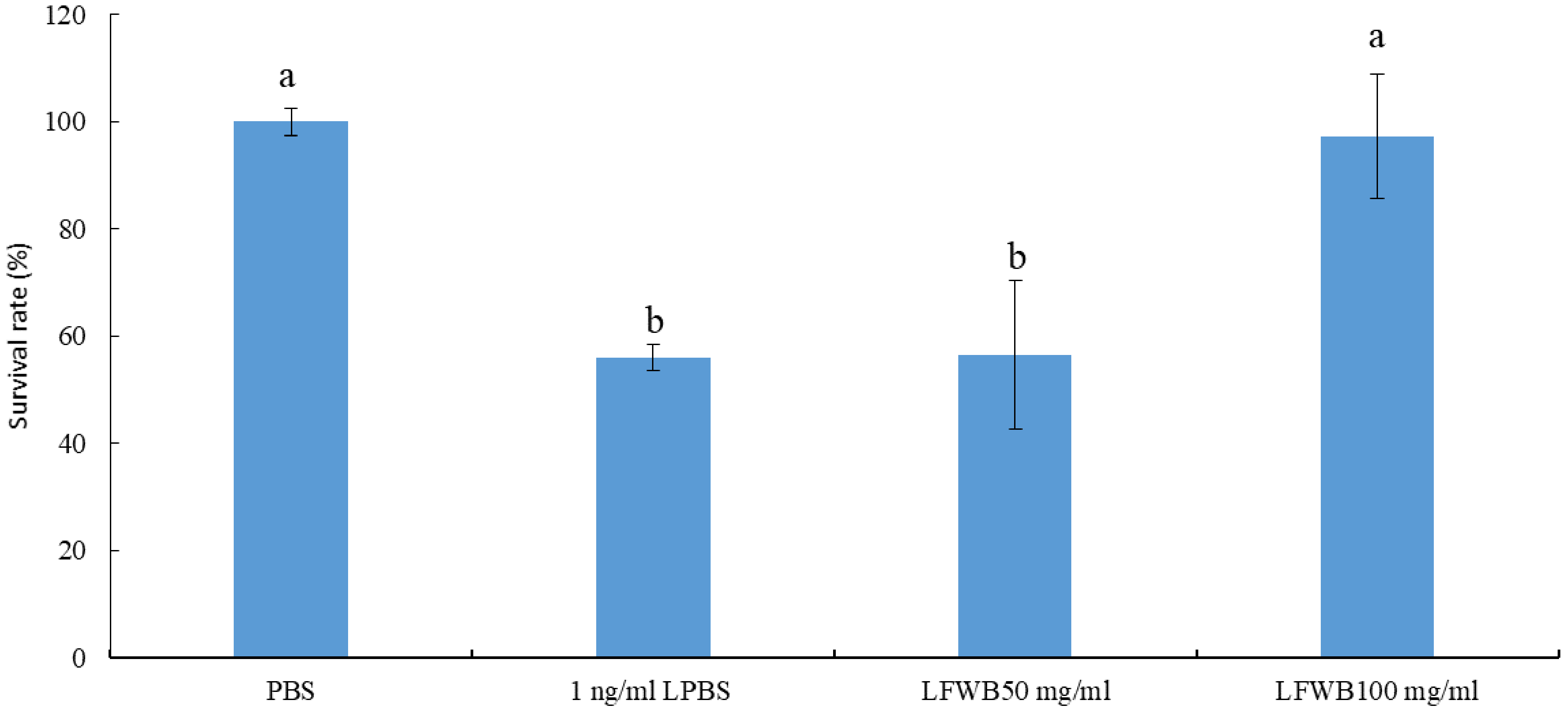

Supplement figure 3

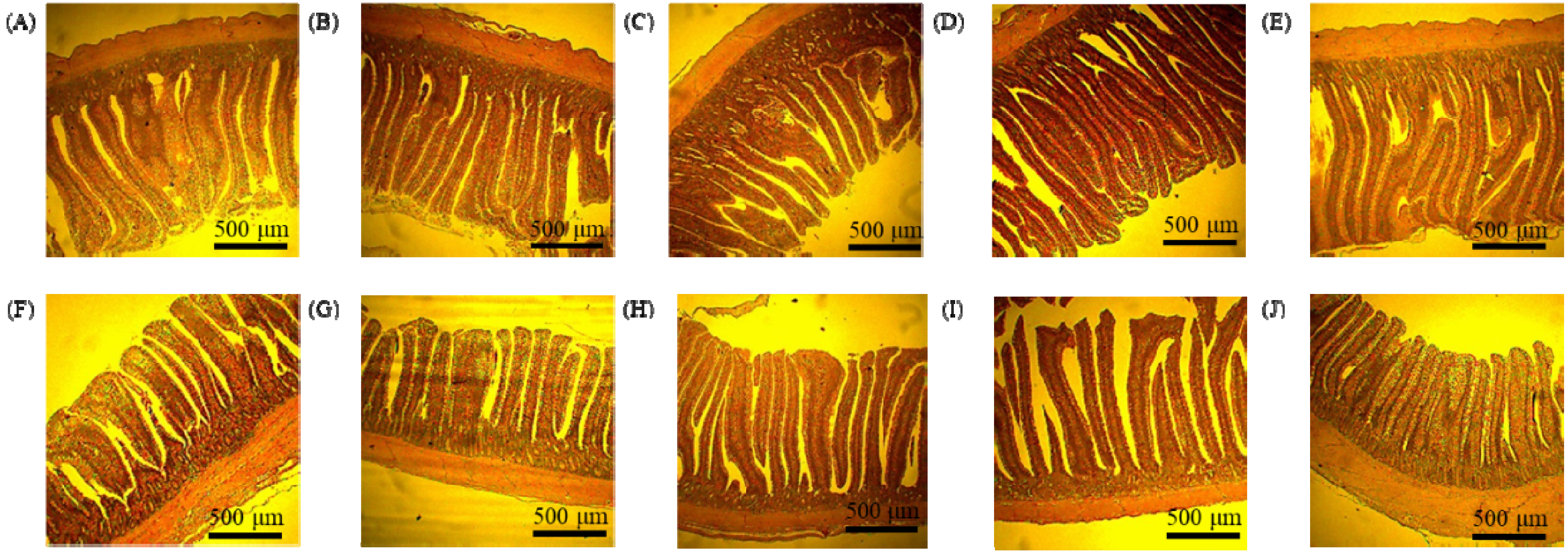

Supplement figure 4

Supplement table 1. The primer sequence of each gene according to genbank.

Gene name[1]	Primer sequence	Genbank No.
ß-actin	F: 5’-CTGGCACCTAGCACAATGAA-3’	X00182.1
	R: 5’-ACATCTGCTGGAAGGTGGAC-3’	
MUC-2	F: 5’-GCTACAGGATCTGCCTTTGC-3’	JX284122.1
	R: 5’-AATGGGCCCTCTGAGTTTTT-3’	
Occludin	F: 5’-GTCTGTGGGTTCCTCATCGT-3’	NM_205128.1
	R: 5’-GTTCTTCACCCACTCCTCCA-3’	
Claudin-1	F: 5’-GGAGGATGACCAGGTGAAGA-3’	NM_001013611.2
	R: 5’-TCTGGTGTTAACGGGTGTGA-3’	
ZO-1	F: 5’-AGGTGAAGTGTTTCGGGTTG-3’	XM_015278975.1
	R: 5’-CCTCCTGCTGTCTTTGGAAG-3’	
NOX-1	F: 5’-CAATGCAGCACTCCACTTTG-3’	NM_001101830.1
	R: 5’-GACAAGATCTCCGCAAGACC-3’	
Nrf-2	F: 5’-GGAAGAAGGTGCGTTTCGGAGC-3’	NM_205117.1
	R: 5’-GGGCAAGGCAGATCTCTTCCAA-3’	
HO-1	F: 5’-GGTCCCGAATGAATGCCCTTG-3’	NM_205344.1
	R: 5’-ACCGTTCTCCTGGCTCTTGG-3’	
GCLC	F: 5’-CAGCACCCAGACTACAAGCA-3’	XM_419910.3
	R: 5’-CTACCCCCAACAGTTCTGGA-3’	
IL-6	F: 5’-AGGACGAGATGTGCAAGAAGTTC-3’	NM_204628
	R: 5’-TTGGGCAGGTTGAGGTTGTT-3’	
iNOS	F: 5’-TACTGCGTGTCCTTTCAACG -3’	U46504
	R: 5’-CCCATTCTTCTTCCAACCTC-3’	
NFκB	F: 5’- CCAGGTTGCCATCGTGTTCC- 3’	D13719.1
	R: 5’- GCGTGCGTTTGCGCTTCT -3’	
COX-2	F: 5’-TGTCCTTTCACTGCTTTCCAT-3’	NM_001167718.1
	R: 5’-TTCCATTGCTGTGTT TGAGGT-3’	
IL-1ß	F: 5’-GCTCTACATGTCGTGTGTGATGAG-3’	NM_204524
	R: 5’-TGTCGATGTCCCGCATGA-3’	
IFN-γ	F: 5’-CTCCCGATGAACGACTTGAG-3’	Y07922
	R: 5’-CTGAGACTGGCTCCTTTTCC-3’	

[1] MUC2: Mucin2; ZO-1: Zonula occludens; NOX-1: NADPH oxidase-1; Nrf-2: Nuclear factor erythroid 2–related factor 2; HO-1: Heme oxygenase-1; GCLC: Glutamate-cysteine ligase catalytic; IL-6: Interleukin-6; iNOS: Inducible nitric oxide synthases; NFκB: Nuclear factor kappa B p 65; COX-2: Cyclooxygenase-2; IL-1ß: Interleukin-1ß; IFN-γ: Interferon-γ.

Supplement table 2. The enzyme activities, carbohydrate and proximate analysis of *Saccharomyces cerevisiae* and phytase co-fermented wheat bran (FWB).

	Products	
	WB[1]	FWB[2]
Enzymes	-----------------------------U/g DM-----------------------------	
Xylanase[3]	ND[4]	152.76
Protease[5]	ND	549.23
Cellulase[6]	ND	289.49
ß-glucanase[7]	ND	147.07
Oligosaccharides	----------------------------- mg/g DM -----------------------------	
Pentose	75.3±0.38	193.80±19.99
Hexose	127.4±3.46	297.50±33.12
D-xylose	ND[3]	16.01±1.24
Xylobiose	ND	21.47±1.89
Xylotriose	ND	16.23±3.11
Proximate analysis[8]	reviewer idea:	
DM (%)	91.5±0.02	91.7±0.01
CP (% DM)	17.8±0.84	20.3±1.23
NDF (% DM)	42.4±1.64	44.9±0.16
ADF (% DM)	15.5±0.30	14.7±0.37
Ash (% DM)	5.15±0.08	5.90±0.37
EE (% DM)	4.08±0.02	4.24±0.05

[1] WB: wheat bran.

[2] FWB: *Saccharomyces cerevisiae* and phytase co-fermented wheat bran.

[3] One unit of xylanase activity is defined as 1 μmol D-xylose is generated from 10 mg/ml xylan in the condition of 37°C and pH 5.5 in a minute.

[4]No detectable.

[5]One unit of protease activity is defined as 1 μg L-tyrosine is generated from 10 mg/ml casein in the condition of 40°C and pH 7.5 in a minute.

[6] One unit of cellulase activity is defined as 1 μmol reducing sugar is generated from 10 mg/ml CMC in the condition of 37°C and pH 5.5 in a minute.

[7] One unit of ß-glucanase activity is defined as 1 μmol reducing sugar is generated from 5 mg/ml ß-glucan in the condition of 37°C and pH 5.5 in a minute.

[8] DM: Dry matter; CP: Crude protein; NDF: Neutral detergent fiber; ADF: Acid detergent fiber; EE: Ether extra.
